# Evaluation of the Proximity of the Maxillary Teeth Root Apices to the Maxillary Sinus Floor in Romanian Subjects: A Cone-Beam Computed Tomography Study

**DOI:** 10.3390/diagnostics15141741

**Published:** 2025-07-09

**Authors:** Vlad Ionuţ Iliescu, Vanda Roxana Nimigean, Cristina Teodora Preoteasa, Lavinia Georgescu, Victor Nimigean

**Affiliations:** 1 Department of Oral Rehabilitation, Faculty of Dentistry, Carol Davila University of Medicine and Pharmacy, 8 Barajul Iezeru Alley, 013871 Bucharest, Romania; vlad-ionut.iliescu@drd.umfcd.ro (V.I.I.); lavinia.butincu@umfcd.ro (L.G.); 2 Department of Scientific Research Methodology and Ergonomics, Faculty of Dentistry, Carol Davila University of Medicine and Pharmacy, 2-4 Eforie Street, 050037 Bucharest, Romania; 3 Department of Anatomy, Faculty of Dentistry, Carol Davila University of Medicine and Pharmacy, 8 Eroii Sanitari Blvd., 050474 Bucharest, Romania; victor.nimigean@umfcd.ro

**Keywords:** teeth–sinus relationships, cone-beam computed tomography (CBCT), maxillary posterior teeth, odontogenic maxillary sinusitis

## Abstract

**Background/Objectives**: Among the paranasal sinuses, the maxillary antrum holds unique clinical relevance due to its proximity to the alveolar process of the maxilla, which houses the teeth. This study aimed to evaluate the position of the root apices of the maxillary canines and posterior teeth relative to the maxillary sinus floor in Romanian subjects. **Methods**: Data for the study were retrospectively obtained from cone-beam computed tomography (CBCT) scans. The evaluation considered the pattern of proximity to the sinus floor for each tooth type, comparisons of the sinus relationships of teeth within the same dental hemiarch, as well as those of homologous teeth, and variation in root-to-sinus distance in relation to sex and age. Nonparametric tests were used for statistical analysis, and multiple comparisons were performed using Bonferroni post hoc correction. **Results**: The study included 70 individuals aged 20 to 60 years. The distance to the sinus floor decreased progressively from the first premolar to the second molar, with median values of 3.68 mm (first premolar), 1.45 mm (second premolar), 0.50 mm (first molar), and 0.34 mm (second molar) (*p* < 0.01). Stronger correlations were observed between adjacent teeth than between non-adjacent ones. The distances to the sinus floor were greater on the right side compared to the left; however, these differences were not statistically significant (*p* > 0.05 for all teeth). Concordance between left and right dental hemiarches regarding the closest tooth to the sinus floor was found in 70% of cases (n = 49), most frequently involving the second molars (n = 38; 54.3%). On average, the distance from the sinus floor was smaller in males compared to females, with statistically significant differences observed only for the second molar. Increased age was associated with a greater distance to the sinus floor. **Conclusions**: Of all the teeth investigated, the second molar showed the highest combined prevalence of penetrating and tangential relationships with the maxillary sinus. At the dental hemiarch level, the second molar was most frequently the closest tooth to the sinus floor, and in the majority of cases, at least one posterior tooth was located within 0.3 mm. Accurate preoperative assessment of tooth position relative to the sinus floor is essential when performing non-surgical or surgical root canal therapy and extractions of maxillary molars and premolars. CBCT provides essential three-dimensional imaging that improves diagnostic precision and supports safer treatment planning for procedures involving the posterior maxilla.

## 1. Introduction

The maxillary, frontal, ethmoid, and sphenoid sinuses—paired left/right anatomical structures—together constitute the group known as the paranasal sinuses.

Awareness of the paranasal sinuses dates back to ancient civilizations [1,2]; however, the distinction between the maxillary and frontal sinuses as separate anatomical and functional entities was first documented by Leonardo da Vinci [1].

The maxillary sinus is a cavity within the maxilla that holds unique clinical relevance owing to its proximity to anatomical structures of importance both to otorhinolaryngology and dentistry—the nasal fossa and the alveolar process of the maxilla, respectively. The link between the teeth and the maxillary sinus was recognized by Leonardo da Vinci, who stated in his anatomical writings that “the maxillary sinus contains the humor which nourishes the teeth” [1]. In 1651, Nathaniel Highmore, an English physician and anatomist, provided the earliest comprehensive description and illustration of the maxillary sinus, leading to its designation as Highmore’s antrum [3].

The development of the maxillary sinus begins around the third month of fetal life, and by the fifth month, its growth extends into the surrounding maxilla [4]. Its final stage of growth occurs in conjunction with the eruption of the permanent teeth, typically between the ages of 12 and 14 years, and full development is attained once the adult dentition is complete [4,5]. Hence, the development of the maxillary sinus itself illustrates its close anatomical relationship with the alveolar process of the maxilla, which houses the teeth.

Corresponding to the alveolar process of the maxilla, the floor of the maxillary sinus may form culs-de-sac (alveolar recesses) between adjacent teeth or between individual roots of the maxillary molars—primarily between the roots of the first and the second molars—in approximately 50% of cases [6,7].

The extension of the maxillary sinus into the body of the maxilla is variable, a key factor in understanding the anatomical relationship between maxillary teeth and the sinus floor [8,9]. This proximity has important clinical implications. Odontogenic infection can penetrate and spread into the sinus cavity [5,10,11]. Root canal treatment (particularly due to the risk of instruments or materials passing beyond the apex), apicoectomy, and tooth extraction are dental procedures associated with a high risk of maxillary sinus damage in cases where the teeth are in proximity to the sinus [5,6,12]. Scientific evidence indicates that recurrent acute sinusitis is frequently linked to chronic dental infection or inflammation, which compromises the integrity of the sinus membrane and increases its susceptibility to subsequent rhinogenous bacterial colonization [13,14]. Moreover, it has been demonstrated that odontogenic chronic rhinosinusitis has a notably greater detrimental impact on general health-related quality of life as compared to non-odontogenic cases [10]. The thickness of the bone separating the dental alveoli from the maxillary sinus ranges from 0.5 mm to 4.5 mm, and the thinner the bone is, the more frequently perforations of the sinus floor and mucosal injuries occur during various dental treatments [7]. Therefore, clinicians must be aware of these anatomical relationships and carefully assess the proximity of the sinus floor prior to planning endodontic therapy, endodontic surgery, or tooth extraction [6].

The relationships of the maxillary posterior teeth with the maxillary sinus have been studied in diverse populations, such as those in Israel [15], Turkey [8], Korea [9,16], Saudi Arabia [17,18,19], India [20], China [21,22], Bulgaria [23], Iran [24], Brazil [25,26], etc.

The present research on the position of the maxillary teeth roots relative to the maxillary sinus floor targets subjects from Romania, since they have not been the focus of earlier studies.

## 2. Materials and Methods

### 2.1. Study Design and Patient Selection

The data used in the study were retrospectively obtained from cone-beam computed tomography (CBCT) examinations performed as part of comprehensive diagnostic and treatment planning procedures at a private dental clinic in Bucharest. The study complies with the Declaration of Helsinki (seventh revision, 2013, Brazil) on research involving human subjects [27] and was approved by the Scientific Research Ethics Committee of the Carol Davila University of Medicine and Pharmacy in Bucharest (Protocol Code PO-35-F-03, Approval No. 34657/21 November 2024). The analysis was carried out between 25 November 2024 and 31 March 2025.

Inclusion criteria
  - Patients who provided written informed consent at the time of their radiographic examination allowed for the potential use of their anonymized information in future scientific research publications.
  - Availability of CBCT scans including the maxillary sinuses, or at least their lower third.
  - Dentate adult subjects aged over 20 years.
  - Presence of fully erupted canines, premolars, and first and second molars in the dental arch.
Exclusion criteria
  - Subjects with a diagnosis of chronic maxillary sinusitis.
  - Subjects presenting with chronic apical periodontitis or root resorption affecting the canines, premolars, or first and second molars.
  - History of non-surgical or surgical root canal treatment involving the aforementioned teeth.
  - History of orthodontic treatment or ongoing orthodontic therapy at the time of radiographic data acquisition.

Eligible subjects were included consecutively based on previously acquired CBCT scans. A formal sample size calculation was not conducted, as the study was designed to utilize the full set of available qualifying cases rather than being based on an a priori sample size calculation.

CBCT scans were acquired with a NewTom VGi Evo device (Cefla S.C., Imola, Italy), operating at 110 kV and 1–20 mA, with an X-ray exposure duration of 3.5–4.3 s, a total scanning time of 18 s, and an effective dose of approximately 100 μSv. Image data were then processed using NNT software, version 16.2.

**Figure 1:**
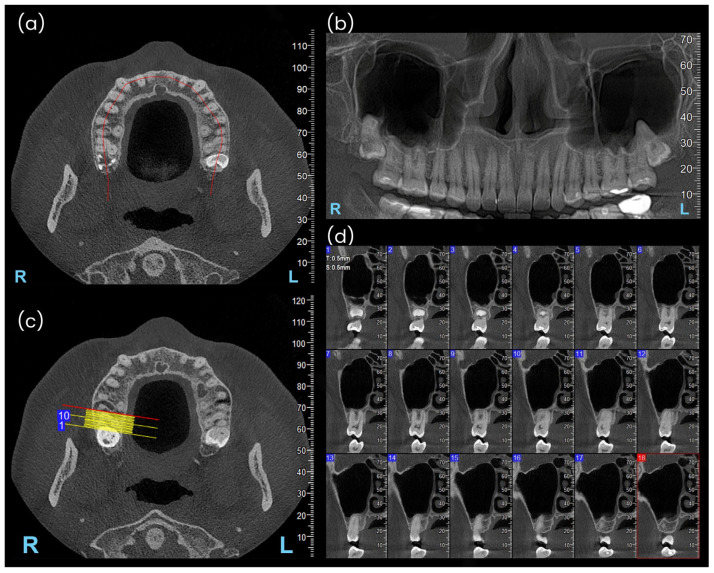
Radiographic data processing algorithm: (**a**) Reference axial image of CBCT used for curved panoramic reformation (red curved line); (**b**) Panoramic reformatted image; (**c**) Setting up buccolingual sections for tooth 17 on the reference axial image; (**d**) Set of serial cross-sectional images perpendicular to the curve of the dental arch and to tooth 17.

An overall assessment—primarily for verifying certain eligibility criteria of the subjects—was performed on a panoramic reformat obtained by following the curved line that runs parallel to the dental arch on the axial image at the cervical area of the teeth (Figure 1a,b). The distances from the root apices to the maxillary sinus floor (vertical anatomical relationships) were evaluated on cross-sectional images with a thickness and interval of 0.5 mm, obtained through the maxillary alveolar process and teeth (Figure 1c,d).

The FDI World Dental Federation notation system was used for teeth identification.

Measurements, expressed in millimeters, were performed on CBCT images at a 1:1 scale. For multi-rooted teeth—the first premolar and the first and second molars—only the relationship of the root closest to the maxillary sinus floor was considered.

**Figure 2:**
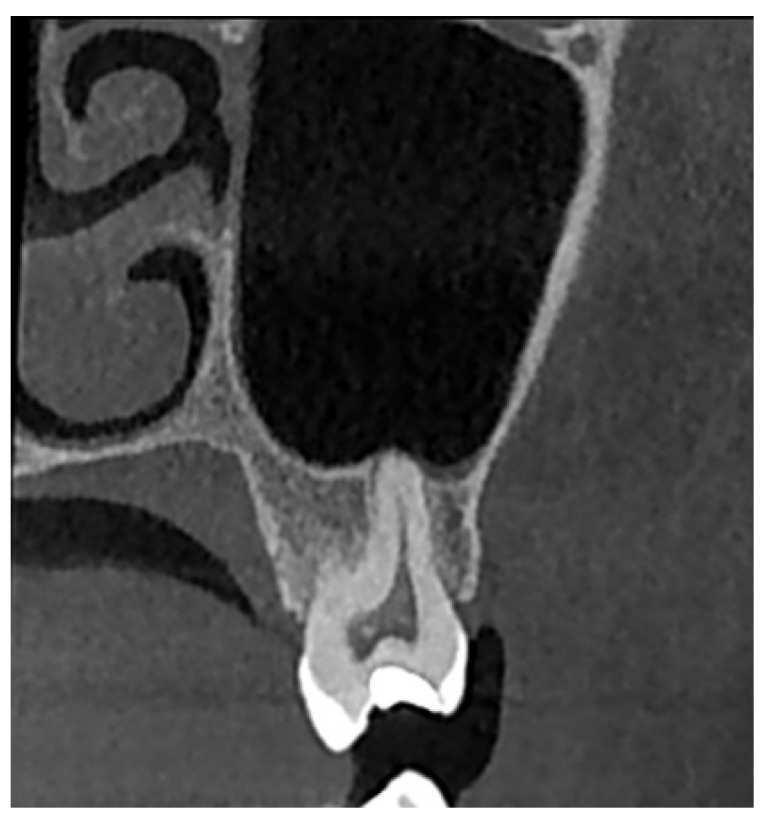
Cross-sectional CBCT reformat along the long axis of tooth 27. Penetrating-type relationship between tooth 27 and the maxillary sinus.

**Figure 3:**
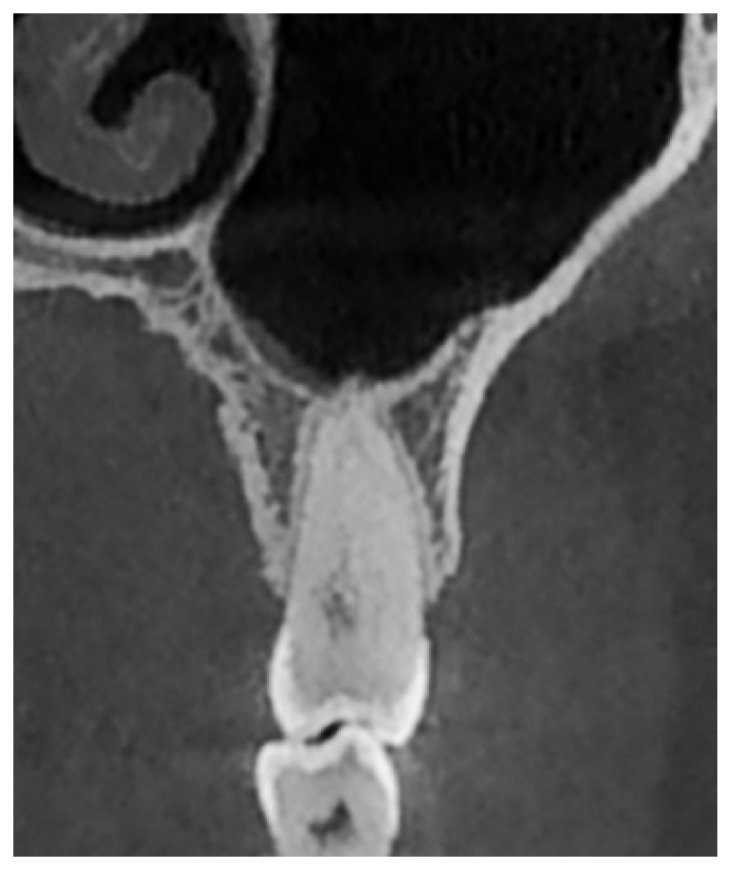
Cross-sectional CBCT reformat along the long axis of tooth 25. Tangential-type relationship between tooth 25 and the maxillary sinus.

**Figure 4:**
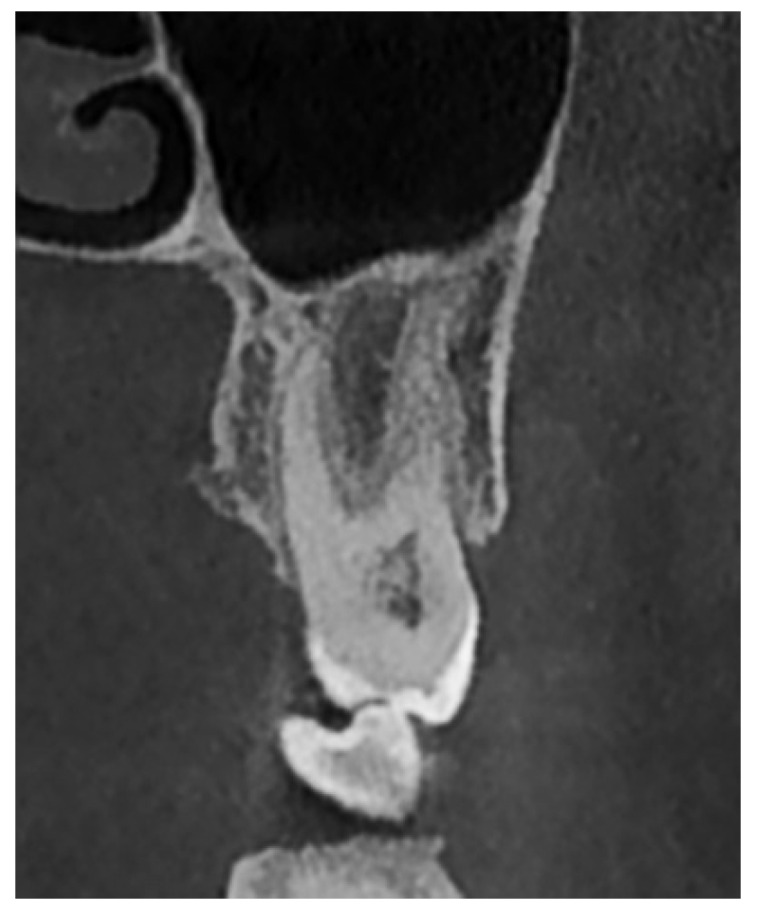
Cross-sectional CBCT reformat along the long axis of tooth 27. Close-type relationship between tooth 27 and the maxillary sinus.

**Figure 5:**
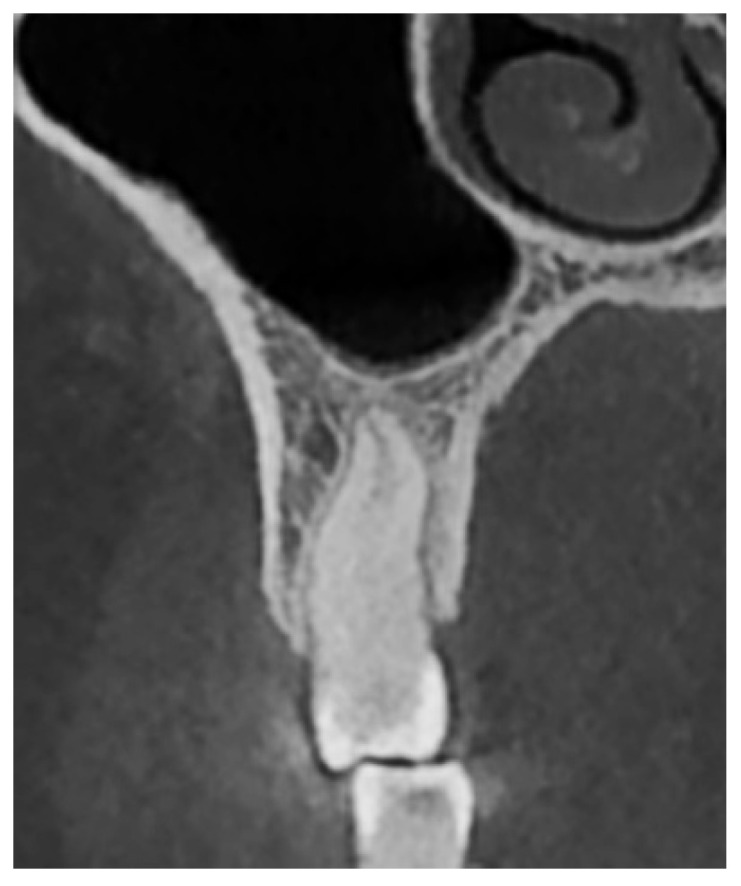
Cross-sectional CBCT along the long axis of tooth 15. Spaced-type relationship between tooth 15 and the maxillary sinus.

The evaluation of the vertical relationship between the teeth and the maxillary sinus was primarily based on the classification proposed by Nimigean et al. (2008), which defines three types: penetrating, tangential, and spaced [28]. For the purposes of this study, the original classification was modified and adapted as follows:

- Type I: Penetrating relationship—the tooth root extends into the maxillary sinus cavity (Figure 2).
- Type II: Tangential relationship—the tooth root is located 0–0.20 mm from the sinus floor (Figure 3).
- Type III: Close relationship—the tooth root is located 0.21–2 mm from the sinus floor (Figure 4).
- Type IV: Spaced relationship—the tooth root is located more than 2 mm from the sinus floor (Figure 5).

To ensure consistency and reliability in the measurement process, all CBCT evaluations were conducted by a single examiner with clinical experience and routine practice in CBCT interpretation. To assess intra-examiner consistency, 20% of the cases were randomly selected and re-evaluated two weeks after the initial measurements. The repeated assessments confirmed the stability of the recorded distances between root apices and the sinus floor, as well as the consistent application of the classification system used. No relevant discrepancies were observed between the initial and follow-up measurements, supporting the reliability of the examiner’s evaluations.

### 2.2. Statistical Analysis

To describe the study participants, categorical variables were summarized using absolute and percentage frequencies, while numerical variables were characterized by indicators of central tendency and dispersion. Data analysis was conducted according to the variable type, and for numerical variables, their distribution pattern was also considered. The main directions of statistical analysis focused on group comparisons and correlation assessment. As the data were not normally distributed, nonparametric tests were applied: the Fisher–Freeman–Halton exact test, Mann–Whitney U test, Spearman correlation test, Friedman test, Wilcoxon test, and Kruskal–Wallis test. Multiple comparisons were performed using Bonferroni post hoc corrections. Statistical analysis was conducted using IBM SPSS Statistics, version 29 (IBM Corp., Armonk, NY, USA). The level of statistical significance was set at *p* ≤ 0.05.

## 3. Results

### 3.1. Sample Description

The study included 70 individuals aged between 20 and 60 years, with a mean age of 40.77 years. The gender distribution was nearly equal, with 34 men and 36 women. The evaluation primarily focused on the premolars and the first and second molars. Only 8 canines (5.71%) were identified in proximity to the maxillary sinus floor. Single-tooth edentulous spaces were of minimal occurrence and were registered for the first premolar (3 subjects), the second premolar (1 subject), and the first molar (2 subjects). In total, 562 maxillary teeth were analyzed across the 70 subjects: 8 canines, 137 first premolars, 139 second premolars, 138 first molars, and 140 second molars.

### 3.2. Relationship of Teeth to the Maxillary Sinus Floor

Evaluating the distance between root apices and the sinus floor revealed the following findings (Table 1):

- Canines most frequently exhibited a spaced relationship with the maxillary sinus floor.
- First premolars predominantly had spaced relationships, less frequently close relationships, and very rarely tangential relationships; no penetrating relationships were observed.
- Second premolars most commonly showed close relationships, followed by spaced relationships. Tangential relationships were observed less frequently and penetrating relationships were not encountered.
- First molars most frequently exhibited close relationships with the sinus floor. Tangential relationships followed in frequency, and penetrating relationships were the rarest. No spaced relationships were observed.
- Second molars most frequently displayed close relationships with the sinus floor, followed by tangential relationships. Penetrating and spaced relationships occurred much less frequently.
- Penetrating and tangential relationships were observed in first and second molars, with their combined frequency increasing with distal positioning of the tooth in the dental arch. Penetrating relationships were more commonly associated with the first molars, while tangential relationships were more frequently observed in the second molars.

For the first molar, the mesiobuccal root was most frequently the closest to the sinus floor (24 teeth; 57.1%), followed by the distobuccal root (15 teeth; 35.7%) and the palatal root (3 teeth; 7.1%), among first molars with types I and II relationships.

For the second molar, the mesiobuccal root was most commonly the closest to the sinus floor (30 teeth; 58.8%), followed by the distobuccal root (17 teeth; 33.3%) and the palatal root (4 teeth; 7.8%), among second molars with types I and II relationships.

Further evaluation (Table 2) highlighted a trend indicating that the distance to the sinus floor progressively decreased from the first premolars to the second molars, with statistically significant differences observed between all molars and premolars within the dental hemiarches analyzed. Canines were not included in this assessment due to their very limited number adjacent to the sinus floor.

An analysis was conducted to identify correlations between the absolute values of the distances from the sinus floor, and the following were established (Table 3):

- Statistically significant correlations were observed for all pairs of posterior teeth analyzed.
- Stronger correlations were observed between the distance to the sinus floor of each tooth and that of the adjacent tooth positioned distally.
- The correlation strength between neighboring teeth was higher than between non-neighboring teeth.
- The correlation coefficients between adjacent teeth increased in strength with the distal positioning of the tooth.

**Figure 6:**
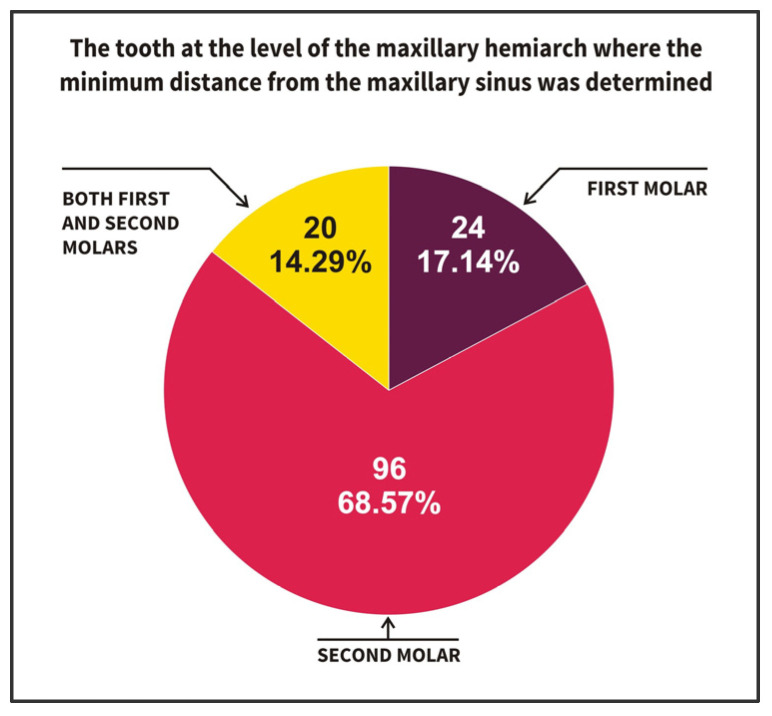
Location of the minimum distance to the sinus floor in relation to tooth type at the level of the dental hemiarch.

Regarding the smallest distance to the sinus floor at the level of each dental hemiarch (Figure 6), this was observed at the level of the second molars in most dental hemiarches (n = 96; 68.57%). Less frequently, it was found at the level of the first molars (n = 24; 17.14%), at both the first and second molars in the remaining cases (n = 20; 14.29%), and never at the level of canines or premolars. The majority of dental hemiarches analyzed presented at least one tooth located less than 0.3 mm from the sinus floor. Specifically, the minimum distance between the root apex and the sinus floor at the dental hemiarch level had a median value of 0.295 mm, with a first quartile of 0.14 mm and a third quartile of 0.77 mm (Table 4).

### 3.3. Relationship with the Maxillary Sinus Floor of Homologous Teeth

When assessing whether there were differences in the type of relationship with the maxillary sinus floor between homologous teeth, no statistically significant differences were observed between the right and left sides (Table 5). The greatest differences were noted for the canines and the first molars. However, the findings related to the canines cannot be reliably interpreted due to the limited number of these teeth included in the analysis. For the first molars, type I and type II relationships with the sinus floor were more frequently observed on the right side than on the left (Table 5).

The distance from the sinus floor of homologous teeth was greater on the right side compared to the left side. However, using the Wilcoxon test, it was observed that this difference was not statistically significant for any of the homologous tooth pairs (Table 6).

Observing the extent of concordance between the left and right sides for the teeth closest to the maxillary sinus floor, the following aspects were noted (Table 7):

- The concordant tooth pairs were the majority, indicating symmetry between the left and right sides for the teeth closest to the sinus floor (referring to 49 concordant pairs, 7 + 38 + 4);
- Among the concordant pairs, the most frequently observed situation (n = 38 concordant pairs) was represented by the left and right second molars (n = 38; 54.3%).
- The number of discordant pairs was 21, with the most frequent situations being
  ○ On the right dental hemiarch, the first molar was the closest tooth to the sinus floor, while on the left dental hemiarch, the second molar was the closest (7 pairs);
  ○ On the right dental hemiarch, the second molar was the closest tooth to the sinus floor, while on the left side, the first and second molars were equally close (7 pairs).

Additionally, an analysis was conducted to determine whether the absolute value of the minimum distance from the teeth to the maxillary sinus floor differs between the left and right dental hemiarches. The Wilcoxon signed-rank test indicated that the minimum distance was statistically significantly smaller on the left dental hemiarch compared to the right (Table 8).

Furthermore, the absolute difference in the distance from the maxillary sinus floor between homologous teeth was assessed to determine whether it significantly deviates from the reference value of zero (0). According to the Wilcoxon signed-rank test, statistically significant differences were found between the absolute distance differences and the reference value (0) for all homologous teeth. Based on the median and mean values, smaller differences were observed in molars and larger differences in premolars (Table 9).

### 3.4. Distance from the Root Apices to the Sinus Floor in Relation to Gender and Age

When comparing the types of relationships between the teeth and the maxillary sinus by reference to gender, a tendency was observed for a smaller distance in male subjects compared to female subjects (Table 10).

As a central tendency, the distance from the sinus floor, recorded by the absolute value, was smaller in males compared to females, but statistically significant differences between the groups were observed only for the second molar (Table 11).

Age-wise analysis of the distance from the sinus floor showed no statistically significant differences in the type of relationship between the teeth and the maxillary sinus, except for the first molar (Table 12). Further analysis revealed statistically significant age-related differences only for molars exhibiting type I and type III relationships (adjusted *p*-value with Bonferroni correction of 0.033). The findings suggest that with increasing age, there is a tendency for a greater distance between the tooth roots and the sinus floor.

No statistically significant correlations were observed between the minimum distance to the maxillary sinus floor at the dental hemiarch level and age (Spearman test: correlation coefficient = 0.139, *p* = 0.101) (Table 13).

The age of the subjects was statistically significantly different for those who recorded the minimum distance at the first molar, second molar, and both molars (first and second), in accordance with the Kruskal–Wallis test, where *p* = 0.024 (Table 14). Subsequent pairwise comparisons using the Bonferroni correction revealed a single statistically significant difference, referring to the fact that subjects for whom the first molar was the closest tooth to the sinus floor had a statistically significantly younger age than those for whom the second molar was the closest, *p* = 0.027 (Table 14).

## 4. Discussion

The present study evaluated the relationships between the roots of maxillary teeth and the maxillary sinus floor in Romanian subjects. The assessment focused on documenting the pattern of proximity to the sinus floor for each tooth type, comparing the sinus relationships of teeth within the same dental hemiarch, as well as those of homologous teeth, and analyzing the variation in root-to-sinus distance with respect to gender and age.

Regarding the first variable analyzed—the relationship to the sinus floor for each tooth type—it was found that first premolars most frequently exhibited a spaced relationship, while second premolars, first molars, and second molars most often showed a close relationship with the maxillary sinus floor.

When summing the types of relationships I-III, they represented 100% for the first molar, 97.1% for the second molar, and 58.7% for the second premolar. Based on these findings, the second premolar, first molar, and second molar can be regarded as teeth in anatomical relationship with the maxillary sinus. The combined frequency of Type I–II relationships (penetrating and tangential) was 7.9% for the second premolar, 30.4% for the first molar, and 36.5% for the second molar. These data support the conclusion that the pattern of proximity to the sinus floor is correlated with the tooth position within the dental arch: the more distally the tooth was located, the closer its root apices tended to be to the sinus floor. Statistically significant differences were found in the distances from the sinus floor between premolars and molars, both across tooth types and in pairwise comparisons (*p* < 0.001).

With regard to the distribution of Type I–II relationships by root category, they were most frequently observed at the mesiobuccal root of the second molar, followed by the mesiobuccal root of the first molar, the distobuccal root of the second molar, and the distobuccal root of the first molar.

The findings of the present study indicated that most of the dental hemiarches examined had at least one posterior tooth positioned less than 0.3 mm from the maxillary sinus floor. No comparable data were identified in the literature reviewed.

The canine typically occupies a neutral position between the maxillary sinus and the nasal fossa. However, in rare cases of increased sinus volume, its root apex may be positioned adjacent to the sinus floor. In the present study, this variant was observed in 8 canines, corresponding to 5.71% of the canines examined. Only Type III and IV relationships were encountered for the canines. Few authors have evaluated the position of the canine root apex relative to the maxillary sinus floor. Oishi et al. (2020) reported that 19.6% of 550 assessed canine roots exhibited contact with the maxillary sinus floor [29]. Khojastepour et al. (2021) examined 600 maxillary sinuses and found that 413 extended to the canine region [30]. In 37 of these sinuses (8.96%), the canine root apex showed either a distance of less than 2 mm to the sinus floor or a contact-type relationship [30]. Similarly, Regnstrand et al. (2021) reported that 20% of the 254 examined canines were located within 2 mm of the sinus floor, while 8% were found to be in direct contact with it [31]. A lower percentage of canines situated near the maxillary sinus was reported by Georgiev et al. (2015) [23], in line with the findings of the present study. When evaluating 245 CBCT scans of the maxilla, 465 of the maxillary sinus, and 960 teeth, the authors identified only 10 canines in proximity to the maxillary sinus, 8 of which were positioned less than 2 mm from the sinus floor [23].

Several classifications have been proposed in the literature to describe the vertical relationships between the teeth and the maxillary sinus floor. Lopes et al. (2016) evaluated these relationships at the level of the maxillary posterior teeth using digital panoramic radiographs and CBCT images, and categorized them into four types: Type 0, where a noticeable distance exists between the root apex and the sinus floor; Type 1, where the root apices are in close proximity to the sinus floor (less than 0.5 mm); Type 2, where the roots appear to project into the sinus, yet are in fact located either medial or lateral to it; and Type 3, where the root apices penetrate the maxillary sinus [32].

The vertical relationships between the teeth and the sinus floor, as evaluated by Kwak et al. (2004), were classified into five types: Type I, where the sinus floor is at a distance from the root apices; Type II, where the sinus floor lies below the line connecting the buccal and palatal root apices, and the roots are tangent to the sinus floor; Type III, where the buccal root apex protrudes into the sinus; Type IV, where the palatal root apex protrudes into the sinus; and Type V, where both buccal and palatal root apices protrude into the sinus [16]. These relationships differ from those reported in the current study. As reported by Kwak et al. (2004), the apex of the palatal root of the first premolar was the farthest from the sinus floor [16], which aligns with the present findings. In their study, the apex of the distobuccal root of the second molar was the closest to the sinus floor [16], whereas in the current work, the apex of the mesiobuccal root of the second molar was found to be the closest.

Kilic et al. (2010), in their study using CBCT, found that the distance between the sinus floor and the root apices was greatest at the level of the first premolar and smallest at the level of the distobuccal root of the second molar [8]. The latter observation contrasts with the findings of the present study.

Tian et al. (2016), in a study on a Chinese population, showed that root extension into the sinus was most prevalent for the palatal root of the first molar and uncommon for the first premolar [21].

Gu et al. (2018), who studied the relationship between maxillary teeth and the sinus floor by CBCT, found that the mesiobuccal roots of the second molars had the smallest distance to the sinus floor (0.8 ± 2.5 mm), followed by the distobuccal roots (1.3 ± 2.7 mm), with statistically significant differences (*p* < 0.05) [33]. They also noted that maxillary molars were positioned closer to the sinus floor than premolars [33], which is consistent with the findings of the present study.

Razumova et al. (2019), using CBCT to study the relationship between the sinus floor and posterior tooth roots in accordance with Kwak’s classification [16], reported that type II relationships were most common for the first and second molars, while type I relationships were more common for premolars [34]. The shortest distance to the sinus floor was recorded for the mesiobuccal root of the second molar [34]. These results match those of the current study.

Junqueira et al. (2020) demonstrated that, in Brazilian subjects, the maxillary second molars had the highest number of roots that either protruded into or were, as they described, “in close contact” with the maxillary sinus [26]. These findings align with the present study, and the authors have concluded that second molars require special attention during endodontic treatment and oral surgery procedures [26].

Pei et al. (2020), in a study conducted on a Chinese population, showed that the mesiobuccal root of the maxillary second molar was the nearest to the sinus floor [22], a finding that corresponds with the present study. However, the most prevalent type of relationship observed by the authors in the examined teeth was the absence of contact between the roots and the inferior border of the maxillary sinus [22], which contrasts with the findings of the present study.

Shaul Hameed et al. (2021) [18], in a CBCT study of a Saudi population, reported that the distobuccal root of the second molar had the shortest mean distance from the sinus floor (0.68 ± 0.39 mm), with Kwak’s type II relationships [16] being the most frequently observed. This contrasts with the current research, in which the mesiobuccal root of the second molar was most commonly found to be closest to the sinus floor.

Altaweel et al. (2022) observed that the mesiobuccal root of the second molar was the closest to the sinus floor, while the buccal root of the first premolar was the farthest, with the differences being statistically significant [35]. These findings closely match those of the present work.

Abdulwahed et al. (2023) used CBCT to examine the relationship between maxillary posterior teeth and the sinus floor and found that the mesiobuccal root of the second molar was frequently closest to the sinus floor [19], consistent with the findings of the current research.

Akotiya et al. (2024), in a CBCT study on an Indian population using Kwak’s classification [16], identified the type I relationship as the most common among maxillary posterior teeth, followed by type II [20]. The authors concluded that the mesiobuccal root of the second molar and the root of the second premolar were closest to the sinus floor [20]. These results are only partially consistent with the present findings.

Sarilita et al. (2024), by their research using computed tomography (CT) scans, showed that the penetrating relationship was most frequently observed for the palatal root of the maxillary first molar (20% among all roots of this type), and the contact-type relationship was most common for the mesiobuccal root of the maxillary second molar (18% of all roots tangent to the sinus floor) [36]. These findings are in agreement with those of the current work.

For the second variable analyzed—the position of homologous teeth relative to the sinus floor—this study examined the distribution of relationship types between homologous teeth, assessed left/right concordance in terms of which tooth was closest to the sinus floor, and investigated whether significant differences existed in the minimum distances from the homologous teeth to the sinus.

No statistically significant differences were found between homologous teeth regarding their types of relationship with the maxillary sinus floor. Notably, in first molars, Type I and Type II relationships were more frequently observed on the right dental hemiarch compared to the left. Although the mean distance from the sinus floor was greater on the right dental hemiarch than on the left, this difference was not statistically significant (*p* > 0.05 for all teeth). In the majority of cases (n = 49; 70%), concordance was observed between the dental hemiarches concerning which teeth were closest to the sinus floor, with the highest frequency of concordance found for the left and right second molars (n = 38; 54.3%). Regarding the minimum distance between the analyzed teeth and the floor of the maxillary sinus, this was found to be statistically significantly smaller on the left dental hemiarch compared to the right (*p* < 0.05). To the best of our knowledge, this is the first study to investigate all three aspects of symmetry in tooth position relative to the maxillary sinus floor: relationship types, the teeth closest to the sinus floor, and the minimum distance. Most available evidence suggests no statistically significant differences between the left and right sides in the position of tooth root apices relative to the sinus floor [8,24,33,34,35,37]. Junqueira et al. (2020) found no statistically significant differences between the left and right sides regarding the distances of homologous root apices to the sinus floor [26]. However, the frequency of the penetrating-type relationship for the mesiobuccal root of the second molar was higher on the left side (37 roots) compared to the right side (28 roots) [26]. Akotiya et al. (2024) reported differences between the sides, noting that the greatest distance to the sinus floor was recorded for the left first premolar (8.24 mm), while the smallest distance was observed for the mesiobuccal root of the left second molar (1.35 mm) [20]. Pei et al. (2020) showed that, in most cases, the distances from posterior maxillary teeth to the sinus floor were shorter on the left side than on the right; however, these differences were not statistically significant [22].

In the current study, sex-wise analysis showed that the distance between tooth root apices and the sinus floor was smaller in male subjects compared to their female counterparts. However, statistically significant differences between sexes were observed only for the second molars.

When the distances were analyzed in relation to age, no statistically significant differences were found, except for molars with Type I and Type III relationships.

Few studies have specifically examined the relationship between teeth and the maxillary sinus in relation to sex and age. Altaweel et al. (2022) reported that sex did not significantly affect the distance between the roots of posterior teeth and the sinus floor [35]. Contrary to their findings, a tendency for shorter distances in male subjects was observed in the present study. Altaweel et al. also noted a significant increase in this distance with age [35], a trend that was also observed in the current research.

Kilic et al. (2010) found no statistically significant differences between male and female subjects in their Turkish cohort [8]. Similarly, Pei et al. (2020) reported that sex did not significantly affect the distance between molar roots and the sinus floor; however, in contrast to the present findings, they observed slightly shorter distances in women than in men, though the difference was not statistically significant [22]. Pei et al. also found a significant age-related increase in the distance from maxillary molar roots to the sinus floor [22].

Von Arx et al. (2014), in a study similar to the present one, reported that premolars were closer to the sinus floor in men than in women, potentially due to men having longer tooth roots and larger maxillary sinuses [38]. They also observed no significant age-related differences in the distance between premolars and the sinus floor [38].

Razumova et al. (2019) found no statistically significant differences related to age in their study on a Russian population [34]. In contrast, Gu et al. (2018) concluded that age significantly affects the relationship between root apices of maxillary posterior teeth and the sinus floor, noting that this distance tends to increase with age [33]. Tian et al. (2016) also demonstrated an age-related pattern, with tooth root apices positioned closer to the sinus floor in individuals under 20 years of age and farther away in those over 60 [21].

An explanation for the age-related increase in the distance from root apices to the sinus floor is the fact that maxillary sinus volume expands until approximately age 20, after which it begins to decrease [39]. This was demonstrated by Ariji et al. (1994) in a study of 115 subjects aged 4 to 94 years, including 61 dentate individuals over the age of 20 [39]. Another possible explanation, proposed by Altaweel et al. (2022), is the phenomenon of physiological tooth eruption, which occurs as a compensatory mechanism for the reduction in clinical crown height associated with aging [35].

In contrast, the study by Ok et al. (2014) found that tooth root penetration into the maxillary sinus increased after the age of 60 [37]. The authors attributed this finding to the common occurrence of adjacent tooth loss in older individuals, which may lead to sinus pneumatization and a subsequent reduction in alveolar bone height [37].

An interesting observation of the present study was that subjects for whom the first molar was the closest tooth to the sinus floor had a statistically significantly younger age compared to those for whom the second molar was the closest. Given that the first permanent molar occupies a strategic position in the dental arch, where it bears the greatest masticatory load [40,41], this finding could be explained by bone apposition over time, a process known to occur in response to mechanical stimulation [42,43]. It has been suggested that such bone apposition occurs between the tooth root apices and the sinus floor [43].

Other authors have examined the correlation between the divergence angle of maxillary molar roots and their proximity to the sinus floor, suggesting that a greater divergence angle is associated with a shorter distance to the sinus floor [44]. This aspect was not evaluated in the current study.

In the present study, CBCT was used to investigate the relationships between the teeth and the maxillary sinus floor. Until approximately two decades ago, orthopantomography (OPG) was the primary radiographic method for evaluating maxillofacial anatomical structures [45]. Amani et al. (2023) indicated that OPG may be a reliable diagnostic tool only when there is a measurable distance between the tooth root apices and the maxillary sinus floor [46]. The same authors emphasized the importance of CBCT evaluation when tooth roots are tangent to, or penetrating into, the sinus [46]. Similarly, Ahmed et al. (2018) highlighted the necessity of CBCT assessment in cases where tooth root projection into the maxillary sinus is observed on OPG images [47]. Rodriguez et al. (2024) compared pairs of OPGs and CBCT scans performed on 28 patients and concluded that panoramic radiography is not a reliable method for assessing the proximity of tooth root apices to the maxillary sinus floor [45]. Other researchers showed that CBCT should be preferred over OPG for accurate evaluation of sinus involvement risk during surgical procedures on the maxillary posterior teeth [48]. Jung et al. (2020) showed that the depiction of tooth root apices within the maxillary sinus on panoramic radiographs demonstrated only moderate accuracy in predicting whether roots actually extended into the sinus [49]. Their study highlighted that disruption of the sinus floor contour on panoramic images may indicate tooth root intrusion into the sinus [49]. Notably, root protrusion most frequently occurred at the mesiobuccal roots of the second molars [49]. Chaves et al. (2022) emphasized that CBCT is more effective than OPG in evaluating complex anatomical relationships, due to its high spatial resolution [50]. Morgan et al. (2023) further supported the superiority of CBCT, citing its capacity for three-dimensional visualization and multiplanar reconstruction of anatomical structures [51].

The close anatomical relationship between the maxillary posterior teeth and the maxillary sinus helps explain the high incidence of maxillary sinusitis of odontogenic origin—recently reported to account for 30–40% of chronic maxillary sinus inflammation cases [12]. Kuligowski et al. (2021) demonstrated an inversely proportional relationship between the distance from the tooth root apex to the sinus floor and the thickening of the Schneiderian membrane lining the sinus [52]. Alghofaily et al. (2023) identified the second molar as the tooth most frequently associated with Schneiderian membrane thickening, attributing this finding to the proximity of its root apices to the maxillary sinus floor [53]. Kwiatkowska et al. (2022) reported the following teeth as the most common causes of odontogenic maxillary sinusitis: first molar (42.6% of cases), second molar (27.9%), second premolar (19.1%), and canine (1%) [54]. In a 2006 study, Nimigean et al. also emphasized the impact of dental pathologies on sinus health, identifying the second molar (32.4%), first molar (30.6%), second premolar (23.7%), third molar (6.8%), first premolar (5.6%), and canine (0.9%) as contributing teeth [55]. These findings collectively underscore the importance of accurately assessing the spatial relationship between the roots of maxillary molars and premolars and the sinus floor as a critical step in diagnosis and treatment planning in the posterior maxilla.

The inclusion/exclusion criteria applied in qualifying subjects for this study represent a key methodological benefit. This approach allowed for a reliable assessment of the tooth–sinus relationships by eliminating potential confounding factors. The distances between root apices and the maxillary sinus floor were not influenced by apical pathologies, prior interventions, or bone resorption/apposition associated with orthodontic treatment, thereby enhancing internal validity. However, this approach inherently limits the external validity of the findings, as routine clinical practice encompasses a broader range of pathological conditions and complexities. Consequently, while the anatomical relationships documented here are highly accurate, caution is warranted when extrapolating these results to clinical scenarios characterized by periapical tissue inflammation, alveolar bone remodeling due to orthodontic treatment or following adjacent tooth loss, or sinus involvement. Employing CBCT data for the measurements brings yet another notable strength to the research methodology, ensuring a high level of accuracy. Although our study focused on healthy anatomical conditions, the data can serve as a baseline for clinicians when interpreting CBCT scans and estimating the risk of anatomical variations or proximity-related complications. Furthermore, comparison with existing studies conducted in different populations reveals both shared anatomical patterns and ethnic variations, underlining the importance of region-specific data in evidence-based treatment planning.

Despite being a unique study within the Romanian population, certain limitations should be acknowledged. The relatively small number of participants, while adequate for an initial evaluation, might not fully capture the characteristics of the broader population. Expanding the sample size could enhance the generalizability of the findings. In the case of multi-rooted teeth, only the root apex closest to the sinus floor was recorded. This approach was selected based on clinical relevance, as proximity to the sinus is a critical factor in the risk of sinus complications following both root canal therapy and tooth extraction. Future studies should aim to incorporate a broader spectrum of clinical cases presenting with significant pathology to better reflect the diagnostic and therapeutic challenges of maxillary sinus-related dental treatment planning. Additionally, the third molar was excluded from this study due to its morphological and topographical variability. However, its relationship with the sinus floor is of particular clinical interest and will be addressed in forthcoming research.

## 5. Conclusions

In this relatively small Romanian cohort, the pattern of proximity to the maxillary sinus floor was found to correlate with tooth position within the dental arch. Among all teeth examined, the second molar exhibited the highest combined frequency of penetrating and tangential relationships with the maxillary sinus. On average, the distance from the sinus floor was smaller in males compared to females, while increased age was associated with a greater distance. The majority of dental hemiarches included at least one posterior tooth located less than 0.3 mm from the sinus floor.

These findings highlight the clinical importance of accurately assessing root–sinus relationships when planning procedures involving maxillary premolars and molars. However, caution is advised when applying these results to complex clinical situations, where pathological conditions may significantly alter the anatomical relationships observed in healthy cases.

CBCT provides essential three-dimensional imaging that overcomes the limitations of two-dimensional techniques such as OPG, which lacks depth perception and may misrepresent the true spatial relationship between root apices and the sinus. This supports the role of CBCT as a valuable tool in ensuring safe and effective diagnosis, risk assessment, and treatment planning in the posterior maxilla.

## Figures and Tables

**Table 1:** Distance from the maxillary sinus floor based on anatomical relationship for each tooth type.

Tooth	Distance			
	Type I (Penetrating Relationship)	Type II (Tangential Relationship)	Type III (Close Relationship)	Type IV (Spaced Relationship)
Canine	n = 0; 0%	n = 0; 0%	n = 3; 37.5%	n = 5; 62.5%
1st Premolar	n = 0; 0%	n = 1; 0.7%	n = 32; 23.4%	n = 104; 75.9%
2nd Premolar	n = 0; 0%	n = 11; 7.9%	n = 72; 51.8%	n = 56; 40.3%
1st Molar	n = 9; 6.5%	n = 33; 23.9%	n = 96; 69.6%	n = 0; 0%
2nd Molar	n = 4; 2.9%	n = 47; 33.6%	n = 85; 60.7%	n = 4; 2.9%

**Table 2:** Comparisons between the proximities to the maxillary sinus floor of different tooth types.

Tooth	Distance to the Sinus Floor Median (Interquartile Range)	*p*-Value ^1^	*p*-Value ^2^		
			2nd Premolar	1st Molar	2nd Molar
Canine	2.93 (1.04; 5.79) mm				
1st Premolar	3.68 (2.05; 5.13) mm	<0.001	<0.001	<0.001	<0.001
2nd Premolar	1.45 (0.62; 2.54) mm			<0.001	<0.001
1st Molar	0.50 (0.20; 0.15) mm				0.002
2nd Molar	0.34 (0.15; 0.80) mm				

^1^—Friedman Test; ^2^—pairwise comparisons with Bonferroni correction.

**Table 3:** Correlations between the distances to the sinus floor of different teeth.

		1st Premolar	2nd Premolar	1st Molar	2nd Molar
**Canine**	Correlation coefficient	0.810	0.536	0.886	0.759
	*p*	0.015	0.215	0.019	0.029
	n	8	7	6	8
**1st Premolar**	Correlation coefficient		0.682	0.483	0.444
	*p*		<0.001	<0.001	<0.001
	n		134	129	135
**2nd Premolar**	Correlation coefficient			0.724	0.606
	*p*			<0.001	<0.001
	n			127	133
**1st Molar**	Correlation coefficient				0.840
	*p*				<0.001
	n				129

Spearman Test.

**Table 4:** Minimum distance from the sinus floor at the level of the dental hemiarch.

Indicator		Minimum Distance
Mean		0.56
Median		0.295
Minimum		0.00
Maximum		3.02
Quartile	1	0.14
	2	0.295
	3	0.77

**Table 5:** Distance from the maxillary sinus floor of homologous teeth—recorded by relationship types.

Tooth	Side	Type I Relationship	Type II Relationship	Type III Relationship	Type IV Relationship	*p*-Value
Canine	Right	n = 0; 0%	n = 0; 0%	n = 1; 20%	n = 4; 80%	0.464
	Left	n = 0; 0%	n = 0; 0%	n = 2; 66.7%	n = 1; 33.3%	
1st Premolar	Right	n = 0; 0%	n = 0; 0%	n = 17; 24.6%	n = 52; 75.4%	0.572
	Left	n = 0; 0%	n = 1; 1.5%	n = 15; 22.1%	n = 52; 76.5%	
2nd Premolar	Right	n = 0; 0%	n = 7; 10%	n = 34; 48.6%	n = 29; 41.4%	0.576
	Left	n = 0; 0%	n = 4; 5.8%	n = 38; 55.1%	n = 27; 39.1%	
1st Molar	Right	n = 6; 8.8%	n = 19; 27.9%	n = 43; 63.2%	n = 0; 0%	0.284
	Left	n = 3; 4.3%	n = 14; 20%	n = 53; 75.7%	n = 0; 0%	
2nd Molar	Right	n = 2; 2.9%	n = 22; 31.4%	n = 45; 64.3%	n = 1; 1.4%	0.738
	Left	n = 2; 2.9%	n = 25; 35.7%	n = 40; 57.1%	n = 3; 4.3%	

Fisher–Freeman–Halton exact Test.

**Table 6:** Comparison of the distances from the sinus floor between homologous teeth.

Homologous Teeth	Right Side (Median)	Left Side (Median)	*p*-Value
Canine	5.58	1.20	0.180
1st Premolar	2.32	0.61	0.078
2nd Premolar	1.24	0.61	0.239
1st Molar	0.15	0.12	0.250
2nd Molar	0.40	0.10	0.250

Wilcoxon Test.

**Table 7:** Left/right concordance between the teeth closest to the maxillary sinus floor.

Concordance Analysis		Minimum Distance from the Sinus Floor on the Left Dental Hemiarch		
		1st Molar	2nd Molar	1st Molar and 2nd Molar the Same
**Minimum Distance from the Sinus Floor on the Right Dental Hemiarch**	1st Molar	7	7	1
	2nd Molar	2	38	7
	1st Molar and 2nd Molar the same	0	4	4

Background shading indicates the concordant left/right tooth pairs closest to the floor of the maxillary sinus.

**Table 8:** Minimum distance from the maxillary sinus floor on the left dental hemiarch versus the right dental hemiarch.

Indicator		Minimum Distance on the Right Dental Hemiarch	Minimum Distance on the Left Dental Hemiarch	*p*-Value
Mean		0.605	0.5076	0.007
Median		0.365	0.280	
Minimum		0.0	0.0	
Maximum		2.93	3.02	
Quartile	1	0.1475	0.120	
	2	0.365	0.280	
	3	0.885	0.690	

Wilcoxon Test.

**Table 9:** Absolute difference in the distance from the sinus floor of homologous teeth.

Homologous Teeth	Absolute Difference in the Distance from the Sinus Floor of Homologous Teeth Compared to the Value of 0 (Left/Right Difference)		*p*-Value
	Median	Mean	
14–24	0.40	0.79	<0.001
15–25	0.31	0.52	<0.001
16–26	0.12	0.23	<0.001
17–27	0.13	0.23	<0.001

One-sample Wilcoxon rank test; reference value = 0 mm. 14: maxillary right first premolar; 24: maxillary left first premolar; 15: maxillary right second premolar; 25: maxillary left second premolar; 16: maxillary right first molar; 26: maxillary left first molar; 17: maxillary right second molar; 27: maxillary left second molar.

**Table 10:** Types of relationships between the teeth and the maxillary sinus compared by subjects’ gender.

Tooth	Sex of the Subjects		Type of Relationship with the Sinus				Total
			Type I	Type II	Type III	Type IV	
Canine	Male	n	0	0	3	2	5
		%	0%	0%	60%	40%	100%
	Female	n	0	0	0	3	3
		%	0%	0%	0%	100%	100%
1st Premolar	Male	n	0	1	21	46	68
		%	0%	1.5%	30.9%	67.6%	100%
	Female	n	0	0	11	58	69
		%	0%	0%	15.9%	84.1%	100%
2nd Premolar	Male	n	0	5	40	22	67
		%	0%	7.5%	59.7%	32.8%	100%
	Female	n	0	6	32	34	72
		%	0	8.3%	44.4%	47.2%	100%
1st Molar	Male	n	4	19	44	0	67
		%	6.0%	28.4%	65.7%	0%	100%
	Female	n	5	14	52	0	71
		%	7%	19.7%	73.2%	0%	100%
2nd Molar	Male	n	1	28	38	1	68
		%	1.5%	41.2%	55.9%	1.5%	100%
	Female	n	3	19	47	3	72
		%	4.2%	26.4%	65.3%	4.2%	100%

**Table 11:** Distance from the sinus floor by reference to subjects’ gender.

Distance from the Sinus (Median)	Male	Female	*p*-Value
Canine	1.2	5.58	0.143
1st Premolar	3.32	4.1	0.054
2nd Premolar	1.3	1.64	0.522
1st Molar	0.39	0.55	0.462
2nd Molar	0.26	0.45	0.038

Mann–Whitney U Test.

**Table 12:** Types of relationship of the teeth with the maxillary sinus by reference to subjects’ age.

Tooth	Age (Median) Corresponding to the Different Types of Relationships with the Maxillary Sinus				*p*-Value
	Type I	Type II	Type III	Type IV	
Canine	-	-	34	34	>0.999
1st Premolar	-	39	40	41	0.951
2nd Premolar	-	45	36	45	0.503
1st Molar	31	40	43	-	0.039
2nd Molar	34	36	43	36	0.536

Kruskal–Wallis Test.

**Table 13:** Correlation between subjects’ age and the minimum distance of the teeth from the sinus floor.

		1st Premolar	2nd Premolar	1st Molar	2nd Molar	Teeth with Minimum Distance at Dental Hemiarch Level
Age (years)	Correlation coefficient	−0.063	0.007	0.044	0.083	0.139
	*p*-Value	0.466	0.933	0.621	0.340	0.101
	Number of teeth	137	139	138	140	140

Spearman Test.

**Table 14:** Minimum distance to the sinus floor in relation to age.

The Level Where the Minimum Distance Was Registered	Age (Median)	*p*-Value ^1^	Pairwise Comparisons	*p*-Value ^2^
1st M	34	0.024	Minimum distance at 1st M—Minimum distance at 2nd M	0.027
2nd M	41.5		Minimum distance at 1st M—Minimum distance the same for 1st M and 2nd M	0.084
Minimum distance the same for 1st M and 2nd M	41		Minimum distance at 2nd M—Minimum distance the same for 1st M and 2nd M	>0.999

^1^—Kruskal–Wallis Test; ^2^—pairwise comparisons with Bonferroni correction. 1st M: first molar; 2nd M: second molar.

## Data Availability

The data supporting the results of this research can be provided upon reasonable request. Privacy issues preclude the public availability of such data.

## Abbreviations

The following abbreviations are used in this manuscript:

CBCT	Cone-beam computed tomography
1st M	First molar
2nd M	Second molar
CT	Computed tomography
OPG	Orthopantomography
